# The Present Treatment of Simple Pleurisy

**Published:** 1892-08-13

**Authors:** 


### THE PRESENT TREATMENT OF SIMPLE PLEURISY.

One school of workers have in recent years tended to consider all forms of pleuritis as tubercular, but, even if we leave out cases of passive hydrothorax, this extreme view remains unproven. However, patients suffering from pleuritis are remarkably good candidates for subsequent tuberculosis, though many certainly escape, and many seem to owe their illness rather to rheumatic and traumatic conditions, and some to the effects of alcoholism. To insist on the tubercular origin in every instance would be as absurd as to insist on it in every peritonitis or arthritis. Indeed, the treatment found good in these applies very largely to affections of the pleural cavities. Sepsis of various kinds has to be watched for, physiological rest and counter irritation in the simple forms, early evacuation and drainage in purulent ones are the chief aims of treatment. In short, the pericardial and the pleural must be regarded as important joint cavities.

An interesting report in the *Therapeutic Gazette* gives the views of the physicians in the chief Paris hospitals on the management of acute pleuritis. We learn from it that Germain Seé uses neither blisters or medicinal treatment; Talaman only rest in the early stages, and then relies on paracentesis if needed; Hayem employs the salicylates; Dieulafoy merely to relieve pain applies small blisters and morphia, and if the effusion is great, draws off the fluid in small quantities by repeated aspirations; Lecorché and Strauss follow the classical methods; Huchard gives a milk diet, with squills and digitalis; Dujardin Beaumetz employs revulsives and large blisters; Faisans finds that wet cupping in an early stage at least relieves the pain, but he, too, relies on paracentesis if effusion remains; while Moutard Martin gives quinine and paints the side with iodine.

On the whole, then, the Paris physicians show a thorough disbelief in the action of drugs, which is shared by a large number of their brethren in this country. We have no drug, they say, which can cut short the inflammatory process; if effusion occurs, and becomes injurious, we must call in the aid of the surgeon to remove it, and, if purulent, to drain the cavity thoroughly. On the other hand, much has been written of late in favour of treatment by the salicylates, under which it is claimed that serous effusions vanish far quicker and more certainly than they would do if left to themselves. So reliable is the method, according to some observers, that they consider it a test that an effusion is purulent if it remains unaffected by a due course of salicylates. Tetz administers one-gramme doses of the sodium salt four to six times on the first day, and then three or four times daily until the effusion disappears. The sooner the treatment is commenced the better, but it is efficacious even with effusions of some weeks' standing.

Still the method is not infallible, and we find the theory advanced that the success of the salicylate plan is seen chiefly in those cases which are of rheumatic origin. Thus a recent author has published a series of instances with a rheumatic history where it was effectual, and others of a tubercular or alcoholic origin where it failed. Though in such a complaint as pleurisy it is difficult to say that a given case would not have recovered without treatment, there seems such a mass of evidence in favour of the salicylates that they will doubtless be frequently employed. If they bring down the temperature without causing sweating, cardiac weakness, or dyspepsia, we are justified in giving them a full trial. Salol has been used in their stead, but without a similar mass of evidence in its favour. Clément praises antipyrin, giving 90 grains in divided doses during the day with marked results, but we should fear marked results of another kind in weakly patients.

In early stages Dr. Vincent Harris employs blisters, painting with iodine, and iodide of potash given internally; while others obtain good results from leeches and mercurial ointment. If non-purulent fluid is present, we can try, without injury to the general health of the patient, seven-grain doses of chloride of sodium every two hours, hydriodic acid, or the syrup of iodide of iron.

Of all remedies against pain, and indeed of all those calculated to prevent effusion, none seems more important than compression by bandages and strips of plaster. The relief afforded by a well-applied bandage is instantaneous and almost magical. In a joint we recognise its value as a matter of daily practice. It is easy to fix a limb on a splint and to apply pressure by a carefully-adapted bandage, but it is difficult to bandage the ribs so as to ensure complete rest for the affected side without embarrassing the respiration; nor does the yielding nature of the lungs permit of such a degree of pressure. If, with care, however, the ribs are bandaged to the opposite shoulder as firmly as possible after a deep expiration, or a horizontal layer of stout strapping be drawn tight across two-thirds of the circumference of the chest in the same manner, relief can be generally obtained, but it is hard matter to do this exactly and thoroughly; either the patient cannot be persuaded to bear it, or the bandage slips up and so becomes useless, or it slips down and impedes the diaphragmatic respiration, which we seek to substitute for thoracic. Still, if we can succeed, the results fully repay us for our trouble. A valuable combination consists in a broad strip of stout belladonna plaster round the side, sewn in front to strong bandages, which are carried round the opposite shoulder and fastened to the plaster at the back. In double pleuritis we must be content with a narrow horizontal bandage, exerting as much compression on the lower ribs of both sides as the patient can bear.

